# Infection Risk Associated With Colonization by Multidrug-Resistant Gram-Negative Bacteria: An Umbrella Review and Meta-analysis

**DOI:** 10.1093/ofid/ofaf365

**Published:** 2025-07-02

**Authors:** Edwin Wilbur Woodhouse, Majd Alsoubani, David J Roach, David B Flynn, Michael LaValley, Kristen Sheridan, David C Hooper, Vance G Fowler, Erin M Duffy, Trudy H Grossman

**Affiliations:** Division of Infectious Diseases, Duke University Medical Center, Durham, North Carolina, USA; Division of Geographic Medicine and Infectious Diseases, Tufts Medical Center, Boston, Massachusetts, USA; Division of Infectious Diseases, Brigham and Women's Hospital, Boston, Massachusetts, USA; Boston University School of Medicine, Boston, Massachusetts, USA; Department of Biostatistics, Boston University School of Public Health, Boston, Massachusetts, USA; Boston University School of Medicine, Boston, Massachusetts, USA; Division of Infectious Diseases, Massachusetts General Hospital, Boston, Massachusetts, USA; Division of Infectious Diseases, Duke University Medical Center, Durham, North Carolina, USA; CARB-X, Boston University, Boston, Massachusetts, USA; CARB-X, Boston University, Boston, Massachusetts, USA

**Keywords:** colonization, infection risk, multidrug-resistant gram-negative bacteria

## Abstract

**Background:**

Infections following colonization of multidrug-resistant gram-negative bacteria (MDR-GNB), particularly Enterobacterales with extended-spectrum beta-lactamases (ESBL-E) or carbapenem-resistant Enterobacterales (CRE), represent a major global health threat. Our aim was to assess quality of evidence and provide estimates on rate of infection following colonization with multidrug-resistant gram-negative bacteria.

**Methods:**

We performed an umbrella review of systematic reviews and meta-analyses. Quality was assessed using the AMSTAR 2 tool, and a meta-analysis was performed to estimate rate of infection.

**Results:**

An initial search for systematic reviews and meta-analyses yielded 847 results, with 17 articles ultimately included. After exclusion of 2 studies for overlapping results and very low quality, the pooled incidence of infection following colonization across the studies was 22% for ESBL-E and 22% for CRE. Few reviews included high-quality findings on mortality or transmission following colonization. Additionally, only a limited number of reviews included findings related to MDR *Pseudomonas aeruginosa* or carbapenem-resistant *Acinetobacter baumannii.*

**Conclusions:**

Our results suggest a substantial rate of infection following colonization of multidrug-resistant gram-negative bacteria. These findings can inform individual patient counseling, future decolonization innovation, clinical trial design, and regulatory approval of new decolonization agents. However, the heterogeneity of the included populations may limit the generalizability of these findings.

## Teaser

Current data on the progression to infection among patients colonized with multidrug-resistant Gram-negative bacteria is highly heterogeneous. We performed an umbrella review and meta-analysis, showing a substantial burden of infection following colonization. This review informs practical benchmarks to enhance methodological practices, facilitate clinical trial design, and compare the efficacy of emerging decolonization strategies.

Infections by antimicrobial-resistant (AMR) bacteria represent a major global health threat, with a high mortality burden worldwide associated with multidrug-resistant gram-negative bacteria (MDR-GNB) [1, 2]. Asymptomatic carriage of bacteria resistant to traditional antibiotics, detected through both culture-based and molecular methods, is thought to be a significant risk factor for the development of infections in colonized individuals [3]. Gastrointestinal colonization with extended-spectrum beta-lactamase–producing Enterobacterales (ESBL-E) and/or carbapenem-resistant Enterobacterales (CRE) is of special concern due to diminishing therapeutic options and associated higher mortality [4].

While there are numerous recognized decolonization interventions to prevent health care–associated infections, they have not shown consistent benefit in clinical trials, prompting research into new decolonization methods [5]. However, more accurate data on adverse clinical outcomes associated with colonization by resistant pathogens are needed to inform clinical trial design of these emerging approaches, in addition to improving infection prevention efforts and patient counseling strategies. Numerous studies in different demographic and geographic cohorts describe an association between colonization with these resistant bacteria and adverse clinical outcomes, including increased infection-related mortality and transmission [6–8]. Several systematic reviews have also been performed on this topic [3, 9–24], but they employed a variety of methods, were conducted in different populations, and reported heterogenous results. As a result, the risk for infection due to colonization with multidrug-resistant gram-negative bacteria is uncertain on a population level.

Umbrella reviews, which perform a systematic review of systematic reviews and meta-analyses on a topic, are a rigorous alternative to traditional systematic reviews to understand and synthesize a large heterogenous field of study. An umbrella review may present a helpful high-level overview of the quality, effect sizes, and heterogeneity of results on a specific topic, but also depends on the quantity and quality of underlying systematic reviews [25]. In the current study, we used an umbrella review approach to evaluate the risk of infection and/or transmission following colonization by MDR-GNB. We aimed to improve population identification, data analysis, and monitoring of MDR-GNB infection following colonization within future observational studies and to benchmark clinical trials of novel decolonization interventions.

## METHODS

We conducted an umbrella review of systematic reviews and meta-analyses. The protocol for this review was preregistered with Open Science Framework (Registration Y9D76).

### Literature Search

Searches were conducted in the electronic databases PubMed, Embase, Web of Science, and Cochrane Database from inception to May 21, 2024, and these searches were repeated before study completion on December 2, 2024. Searches comprised Controlled Vocabulary terms and keywords for Enterobacterales, *Pseudomonas aeruginosa*, and *Acinetobacter baumannii*. Clinically relevant MDR-GNB were defined as extended-spectrum beta-lactamase or carbapenem-resistant Enterobacterales (ESBL-E or CRE), multidrug-resistant *P. aeruginosa* (MDR-PsA), or carbapenem-resistant *A. baumannii* (CRAB). Results were limited to humans, systematic reviews, and meta-analyses. Full search terms can be found in the Supplementary Data. The COVIDence web-based collaboration software platform was used for literature screening and data extraction [26]. Title and abstract screening was performed by at least 2 authors, and disagreements were resolved by consensus with a third author (E.W.W., D.J.R., M.A.). Full-text articles were then screened for final inclusion by 2 authors, and disagreements were resolved by consensus with a third author (E.W.W., D.J.R., M.A.). We included systematic reviews or meta-analyses that described clinical outcomes such as infection, transmission, morbidity, or mortality following colonization with MDR-GNB. Studies that assessed colonization of multiple pathogen types were included, with only data on MDR-GNB analyzed where relevant. Systematic reviews of randomized controlled trials (RCTs) and nonrandomized studies of interventions (NRSIs) were included when rates of colonization leading to infection were clearly described. Studies were excluded if they did not perform a systematic review or meta-analysis, only analyzed outcomes at time of infection, did not differentiate colonization from infection, did not analyze resistant gram-negative bacteria, or did not have an English-text version available. Studies with an abstract only were excluded if the study's corresponding author was unable to provide the full-text study. The patient, intervention, comparison, outcome (PICO) framework for this study can be seen in the Supplementary Data.

### Data Extraction

Data extraction was independently performed by 2 authors, with consensus adjudication performed with a third author to resolve disagreements (E.W.W., D.J.R., M.A.). The following information was extracted: title, contacting author, location of study, study design, methods used, study aim, types of included studies, total number of included studies, start and end dates, organisms included, definition of multidrug resistance, definition of colonization, site(s) of colonization, study funding, author conflicts of interest, types of study participants and comparator group, health care setting, inclusion/exclusion criteria, total number of participants, most common organism, baseline population characteristics, infection incidence after colonization, transmission rate, mortality, risk factors for infection and mortality after colonization, follow-up time, study heterogeneity, quality rating, study bias description, described limitations, and study conclusions.

### Quality Assessment

The AMSTAR 2 critical appraisal tool for systematic reviews was used to perform a quality assessment of all included studies [27]. Component quality rating was performed independently by 2 authors, disagreements were resolved through consensus moderated by a third author (E.W.W., D.J.R., M.A.), and overall quality determination was assigned by consensus score.

### Meta-analysis

Data on clinical outcomes of colonization were grouped by organism in separate categories including ESBL-E, CRE, multidrug-resistant *P. aeruginosa* (MDR-PsA), and carbapenem-resistant *A. baumannii* (CRAB). Studies with a critically low-quality rating on the AMSTAR 2 scale or that did not clearly define resistance type(s) were excluded from the final meta-analysis. To avoid overweighting individual studies, component studies were reviewed within each systematic review to assess for overlap, and systematic reviews containing >50% component-study overlap with other reviews were excluded from the meta-analysis, with inclusion of the highest-quality study when conflict arose. Data were additionally grouped by infection type, either bloodstream infection alone or all sites of infection. Comparator group for risk ratios was defined by each study but generally consisted of risk-matched noncolonized individuals. Summary statistics were generated for each grouping using a random-effects meta-analysis to generate separate forest plots for incidence of infection and risk ratios of infection following colonization. A sensitivity analysis was performed by analyzing all studies regardless of quality assessment in order to estimate possible differences in infection risk driven by critically low-quality studies. Publication bias assessment was performed by visual inspection of the funnel diagrams of estimates plotted against standard errors. Statistical analyses were performed using the *meta* and *metafor* libraries in the *R* software package.

## RESULTS

**Figure 1.**
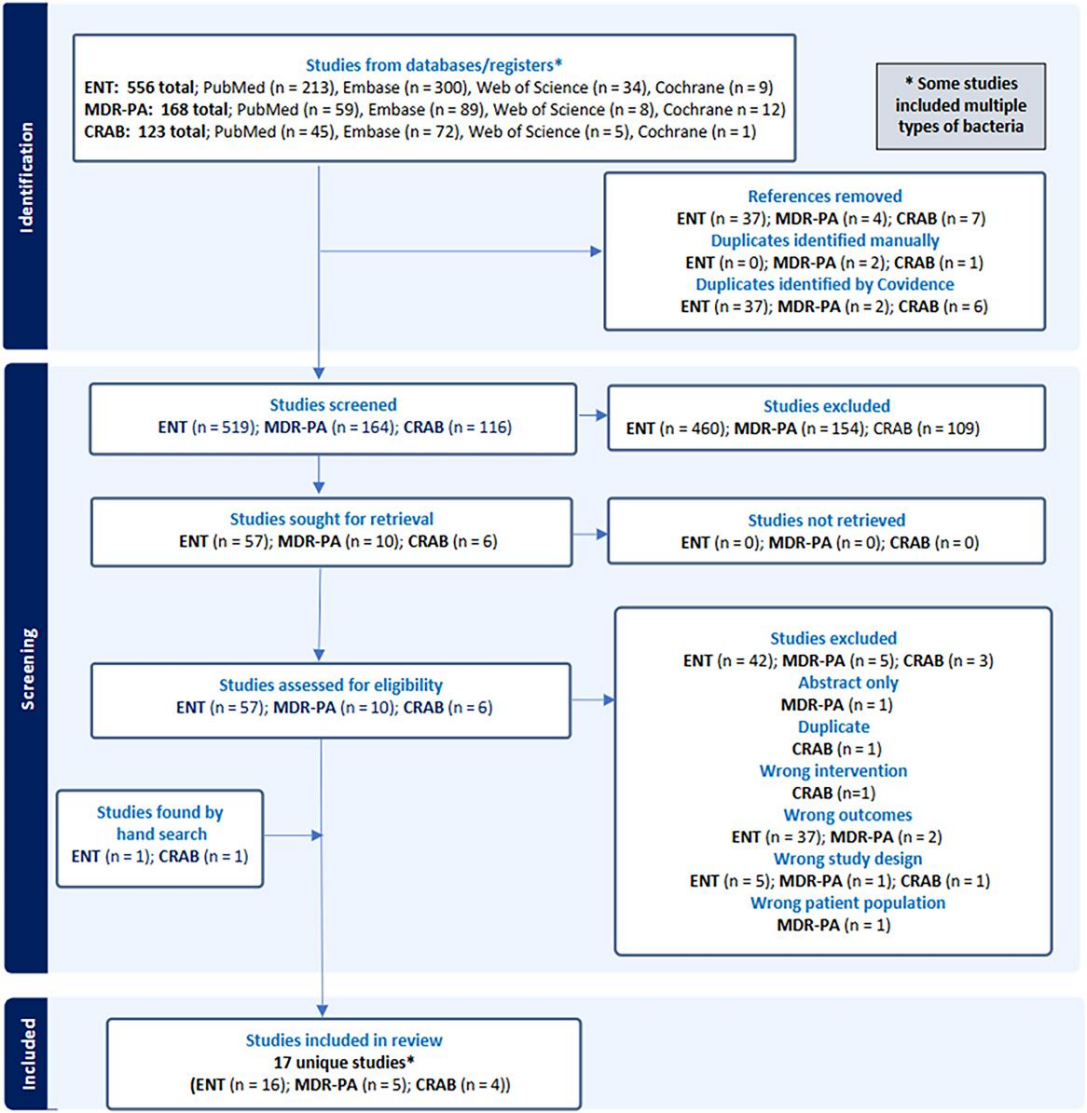
PRISMA flowchart. Abbreviations: CRAB, carbapenem-resistant *A. baumannii*; ENT, resistant Enterobacterales; MDR-PA, multidrug-resistant *P. aeruginosa*.

We identified 847 articles through our initial search of systematic reviews and/or meta-analyses. After removing duplicates and screening titles and abstracts, 73 articles were selected for full text review (Figure 1). The search was updated, including an additional manual search, on December 3, 2024. Of these, 17 articles met the eligibility criteria and were included (Table 1). Notably, only 2 reviews exhibited significant overlap in the primary studies (>50%), with minimal overlap observed among the remaining reviews, and these same 2 reviews also showed critically low quality according to AMSTAR-2 [11, 23]. Visual inspection of funnel plots and evaluation of funding sources of all studies were not suggestive of bias (Supplementary Data).

**Table 1.** Characteristics of Included Reviews

Study and Study Type	Database and Dates of Search	Aim	Organism	Colonization Site	No. of Studies Included	No. of Patients	Patient Population	Inclusion Criteria	Exclusion Criteria	Study Quality (NOS)^a^	AMSTAR 2
Alevizakos 2017 [9] SR/MA	PubMed, EMBASE Feb 1996–Apr 1, 2016	Identify the prevalence of colonization and the relative risk for infection among colonized patients	ESBL-E	Intestinal	4	1089 (adults 1038, pediatrics 51)	SOT recipients	Retrospective and prospective studies with patient-level data; only pre-intervention data; adults and pediatrics; English language only	Case series and case reports; studies that did not differentiate between infection and colonization	All studies were considered high quality	Low
Alevizakos 2016 [10] SR/MA	PubMed, EMBASE Jan 1, 1991–Mar 1, 2016	Estimate the burden of colonization and evaluate the link between colonization and bloodstream infection	ESBL-E	Intestinal	10	2211 (1164 compared colonized vs non)	Adults with hematologic or solid tumor	Gastrointestinal malignancy; microbiological methods included	Non–English language; studies that did not differentiate between infection and colonization	All studies were considered high quality	Critically low
Almohaya 2024 [11] SR/MA	PROSPE, Medline, EMBASE, Wiley Cochrane Library (CDSR and Central), ProQuest Dissertations, Theses Global, SCOPUS Inception–Mar 20, 2023	Assess the risk of mortality and the risk of infection or graft loss in colonized SOT	MRSA, VRE, ESBL, AmpC or CR Enterobacterales, and PsA	Intestinal, respiratory, and/or urine	39 (17 Enterobacterales)	15 202 SOT recipients including 1892 CRE and 548 ESBL-E	SOT recipients	SOT adult recipients who were colonized or received an organ from a donor who was colonized with MDR bacteria within 12 mo pretransplant	MDR colonization with non-lactose-fermenting gram-negatives other than PsA	28/39 were good quality, 2/39 were fair, and 9/39 were poor	High
Arzilli, 2022 [3] SR	PubMed, Cochrane, PsycInfo 2010–Jul 15, 2023	Explore screening practices of MDR GNR and the risk of progression from colonization to infection	MDR PsA, *A. baumannii*, Enterobacterales	Intestinal	62 studies (risk of infection)	8366	Adults inpatient	Studies that provided risk for developing infection during hospital stay among colonized patients	Reports of narrative review, point prevalence studies; case reports and other nonpertinent publication types; pediatric studies; language not English, Spanish, Italian, or French	88 studies were high quality, 8 low quality, and 4 very low quality, Cochrane Risk of Bias Tool for RCTs, NOS score for nonrandomized studies	Low
Bulabula 2020 [12] SR/MA	PubMed, SCOPUS Inception- Mar 31 2019	Review the molecular evidence for transmission of MDR-GNR from colonized mothers to their infants, and summarize factors contributing to transmission	ESBL-E and MDR *Enterobacter cloacae*	Mother rectum, genital and urine, and neonate gastric and vagina	8 in narrative analysis, 6 in meta-analysis	736 mothers, 957 neonates	Mother–infant pairs	Included molecular evidence of transmission	Studies reporting breast milk contamination; outbreaks in neonatal wards; infected mothers	6/8 studies were moderate quality, 2/8 were poor quality	Critically low
Detsis 2017 [13] SR/MA	PubMed, EMBASE Inception- Nov 2015	Assess the ICU acquisition rate of digestive tract colonization with ESBL-E and identify the risk factors for colonization; estimate the risk for subsequent ESBL-E infection	ESBL-E	Intestinal	13	15 045	Adult ICU	Digestive colonization with ESBL-E using double disk synergy test; English language; adults	Studies that did not differentiate between infection and colonization; decolonization interventions; outbreak data	All studies were considered high quality	Low
Ferrer 2022 [14] SR	EDLINE, Cochrane Library, MEDES 2016–2021	Identify the relevant risk factors associated with infection progression	CR PsA, *A. baumannii*, Enterobacterales	Rectal or respiratory	29	NA	Adult inpatients	Patients with respiratory or rectal colonization; language English or Spanish; adults; for respiratory colonization: ICU inpatients with artificial airway	One arm or pre/post studies; studies from low- and middle-income countries; if genetic testing or COVID-19 were included; chronic colonization in the setting of chronic respiratory disease like cystic fibrosis	NA	Critically low
Gao 2024 [15] SR/MA	PubMed, EMBASE, Web of Science Inception– Mar 1, 2023	Examine the risk factors for mortality, colonization, and infection in SOT recipients	CRE	Intestinal	27 in qualitative analysis, 23 in meta-analysis	13 511	SOT recipients	Any study design with patient-level data; adults	Studies that did not differentiate between infection and colonization; reviews, reports, or abstracts; absence of comparator group	All studies were considered high quality	High
Ling 2022 [16] SR/MA	PubMed, EMBASE, SCOPUS Inception–Apr 23, 2021	Understand the transmission dynamics of ESBL-E by estimating duration of carriage in community residents and the rate of transmission within a household	ESBL-E	Intestinal	26	2505	General community residents, discharged hospital patients, and travelers	Studies with index pt with ESBL-E colonization or infection (with known carrier status of household contacts)	Hospital transmission; maternal– neonatal transmission	Modified Joanna Briggs Institute quality assessment tool: limited generalizability of the studies with limited reliability of the outcomes.	Critically low
Margalit 2024 [17] SR/MA	MEDLINE database, Cochrane library, Web of Science, European Congress of Clinical Microbiology and Infectious Diseases, IDWeek libraries, ClinicalTrials.gov	Assess the impact of screening on clinical outcomes including invasive infections and mortality	CRAB	Intestinal (8 studies), respiratory (6 studies), and skin (2 studies)	9 (8 studies reported outcomes)	12 128	Adult ICU and SOT recipients	English language; RCTs and cohort studies; adults with either comparison: (1) screening vs no screening, (2) CRAB carriers and noncarriers	Case–control studies, case series of <20 patients, and case reports	All studies had low risk of bias (NOS 7–9)	High
Martischang 2020 [18] SR	Cochrane Library, PubMed, Embase, CINAHL database Jan 1990–Jun 2018	Assess the co-carriage and acquisition rates of ESBL-E among household contacts	ESBL-E	Intestinal	13	863	Household members of known positive ESBL-E case	Cohort or cross-sectional studies evaluating co-carriage proportions and acquisition rates in household; only human transmission	Studies focusing on international travelers, indigenous populations with a specific way of living, farms, or foodborne community outbreaks; maternal– neonatal transmission	NA	Low
Righi 2023 [19] SR	Medline, Embase, Cochrane database Jan 2010– Apr 30, 2022	Provide evidence-based recommendations for perioperative antibiotic prophylaxis with preoperative MDR-GNR rectal colonization	ESBL-E, CRE, aminoglycoside-resistant Enterobacterales, fluoroquinolone-resistant Enterobacterales, extremely drug-resistant PsA, cotrimoxazole-resistant *Stenotrophomonas maltophilia*, CRAB, colistin-resistant GNR, pan-drug-resistant GNR	Primarily intestinal (1 study had axillary sabs)	32	NA	Adult presurgical patients	Any type of study besides case reports that looked at risk of surgical site infection in adult patients colonized with MDR organisms before surgery	Outpatient studies; studies omitting patients' carrier status before surgery	For uncontrolled studies: 11/26 were high, 11/26 were moderate, and 4/26 were low	Moderate
Tacconelli 2019 [20] SR	PubMed, the Cochrane Database of Systematic Reviews, the Cochrane Central Register of Controlled Trials, Web of Science End Aug 16, 2017	Provide evidence-based recommendations for decolonization of MDR-GNR carriers	ESBL-E, CRE, aminoglycoside-resistant Enterobacterales, fluoroquinolone-resistant Enterobacterales, extremely drug-resistant *Pseudomonas aeruginosa*, cotrimoxazole-resistant *Stenotrophomonas maltophilia*, CRAB, colistin-resistant GNR, pan-drug-resistant GNR	Primarily intestinal (1 study included urine, 1 study included respiratory samples)	27	NA	Mixed population	All studies evaluating any decolonization regimen targeting patients colonized with MDR-GNR; English	Studies involving universal decolonization (decolonization of all patients without previous screening); preoperative surgical prophylaxis; environmental colonization; in vitro and animal studies	The majority of the studies were low to very low quality	High
Tischendorf 2016 [21] SR	PubMed, Medline, Cochrane Library database, Cumulative Index to Nursing and Allied Health Literature, Scielo databases Jan 1, 1991–Jun 2015	Understand the relationship between colonization with CRE and subsequent infection	CRE	Primarily intestinal (2 studies had nares and trachea)	10	1806	Adult inpatients	Clinical trials or observational studies with data provided to calculate rates of infection in patients initially colonized	Studies that did not differentiate between infection and colonization	NA	Critically low
Vink 2020 [22] SR/MA	PubMed End Sep 13, 2019	Collate current evidence on the impact of nosocomial acquisition and transmission of MDR-GNR in hospital settings	MDR Enterobacterales, *E. coli*, PsA, *A. baumannii*	Primarily intestinal (4 studies included oropharyngeal or bronchial samples)	28	27 768	Adult inpatients	Studies that assessed transmission or acquisition of intestinal MDR-GNR in adults within hospital settings	Outbreak settings; studies exclusively assessing the prevalence of MDR-GNR within admitted patients	NA	Critically low
Wang 2024 [23] SR/MA	PubMed, Web of Science, Cochrane Library database Dec 1998–Jun 2023	Investigate the infection rate in rectal carriers and clarify the risk factors that predict subsequent CR *K. pneumoniae* infection in colonized patients	CR *K. pneumoniae*	Intestinal	14	5483	Adult inpatients	Studies that differentiated between infection and colonization; intestinal colonization; incidence rate data available; English language	Reviews, systematic reviews, meta-analyses, editorials, letters to the editor, comments, and case reports; neonatal and pediatric populations	All studies were considered high quality	Critically low
Willems 2023 [24] SR/MA	PubMed, EMBASE, Clarivate Analytics–Web of Science Core Collection Jan 1, 1995–Mar 17, 2022	Incidence of infections caused by resistant bacteria in individuals with enteric or urinary colonization and discern differences in this risk between different patient populations, while taking account of the time at risk	MDR *Acinetobacter baumannii*, MDR PsA, CRE, ESBL-E, VRE	Intestinal	44 total with 40 in the meta-analysis arm; for the GNRs, 29 studies included in the systematic review, 26 studies included in the meta-analysis	9034	Adults	Cohort and case–control studies using incidence-density sampling and including infection outcomes; enteric or urinary colonization; studies that analyzed 50 or more patients with colonization with infections that were clearly preceded by colonization	If time of follow-up was unclear; studies that used incidence proportions or a denominator that involved patients without colonization; RCTs	The quality assessment tool of the NIH was used: the median NIH score (IQR) was 75% (67%–75%), indicating overall higher quality	High

^a^NOS unless otherwise specificized.

Abbreviations: CR, carbapenem-resistant; CRAB, carbapenem-resistant *Acinetobacter baumannii*; CRE, carbapenem-resistant Enterobacterales; ESBL, extended-spectrum beta-lactamase; ESBL-E, extended-spectrum beta-lactamase Enterobacterales; GNR, gram-negative rods; ICU, intensive care unit; MA, meta-analysis; MDR, multidrug-resistant organism; MRSA, methicillin-resistant *Staphylococcus aureus*; NIH, National Institutes of Health; NOS, Newcastle-Ottawa scale; PsA, *Pseudomonas aeruginosa*; SOT, solid organ transplant; SR, systematic review; VRE, vancomycin-resistant *Enterococcus*.

### Evidence Quality

Using the AMSTAR 2 tool, the quality of each study was classified as high, moderate, low, or critically low. Seven reviews were deemed to be of critically low quality, primarily due to the absence of a study protocol and lack of funding description for individual studies included in the review (Table 2). In the included reviews, all but 4 conducted a risk of bias assessment of the studies they examined. Among these, 11 used the Newcastle-Ottawa scale, 1 used the Modified Joanna Briggs Institute quality assessment, and another used the National Institutes of Health assessment tool.

**Table 2.** Meta-analysis Results for Individual Studies

Study	Organism	Infection Ratio (95% CI)	Follow-up Time	Infection Type if Specified
Risk of infection following colonization (incidence)				
Arzilli 2022 [3]	MDR GNR	Incidence 11.0% (8.0%–14.3%)	NA	NA
	Enterobacterales	Incidence 6.8% (4.4%–9.6%)	NA	NA
	*Klebsiella* species	Incidence 18.1% (8.9%–29.3%)	NA	NA
	*A. baumannii*	Incidence 35.3% (0.0%–93.0%)	NA	NA
	*E. coli* (1 study)	Incidence 7.2% (3.0%–12.9%)	NA	NA
	PsA (1 study)	Incidence 56.3% (3.1%–80.0%)	NA	NA
Wang 2024 [23]	CR *K. pneumoniae*	Incidence 23.2% (17.9%–28.5%)	45–90 d	Mixed infection
		Incidence 10% (7%–13.3%)	45–90 d	Bloodstream infection
		Incidence 8% (4%–12%)	45–90 d	Pneumonia
Willems 2023 [24]	MDR-GNR	Incidence density 3.40 (1.87 to 4.92)	NA	Mixed infection
	CRE	Incidence density 4.26 (1.69 to 6.82)	NA	Mixed infection
	ESBL-E	Incidence density 1.82 (0.63 to 3.01)	NA	Mixed infection
	MDR-GNR	Incidence density 2.05 (0.67 to 3.43)	NA	Bloodstream infection
	CRE	Incidence density 2.86 (0.89 to 4.84)	NA	Bloodstream infection
	ESBL-E	Incidence density 1.07 (−0.09 to 2.24)	NA	Bloodstream infection
Risk of infection following colonization compared with noncolonized				
Alevizakos 2017 [9]	ESBL-E	Patients colonized with ESBL-E were 11.74 times more likely to develop an infection with ESBL-E (only 1 study)	4 mo	NA
Alevizakos 2016 [10]	ESBL-E	RR 12.98 (3.91–43.06)	The mean duration (range) of hospitalization, based on 2 studies with 651 patients, was 34.4 (1–159) d	Bloodstream infection
Almohaya 2024 [11]	CRE	OR 19.57 (7.78–49.28)	NA	Mixed infection
	ESBL-E	OR 9.09 (5.59–14.78)	NA	Mixed infection
	MDR PsA (1 study only)	OR 18.88 (4.3–82.8)	NA	Mixed infection
	CRE	OR 22.7 (12.2–42.2)	NA	Bloodstream infection
	ESBL-E	OR 7.42 (4.84–11.37)	NA	Bloodstream infection
Detsis 2017 [13]	ESBL-E	RR 49.62 (20.42–120.58)	Until death or discharge from ICU	Mixed infection
Gao 2024 [15]	CRE	OR 12.91 (5.23–31.88)	NA	Mixed infection
Margalit 2024 [17]	CRAB	OR 11.14 (4.95–25.05)	NA	Mixed infection
	CRAB (3 studies)	OR 16.23 (2.39–110.08)	NA	Bloodstream infection
	CRAB (2 studies)	OR 13.91 (0.20–985.36)	NA	Ventilator-associated pneumonia
Risk of mortality following infection or colonization				
Almohaya 2024 [11]	CRE	OR 3.94 (1.86–8.37)	1 y post-transplant	…
	MDR PsA (1 study only)	OR 3.43 (1.86–8.37)	1 y post-transplant	…
Margalit 2024 [17]	CRAB (colonized vs noncolonized)	OR 1.40 (1.00–1.97)	NA	…
Detsis 2017 [13]	ESBL-E	RR 1.57 (1.25–1.98)	NA	…
Risk of transmission or acquisition				
Bulabula 2020 [12]	ESBL-E and MDR *Enterobacter cloacae*	IR 27% (8%–47%)	NA	…
…	ESBL-E	IR 32% (1%–62%)	NA	…

Abbreviations: CRAB, carbapenem-resistant *Acinetobacter baumannii*; CRE, carbapenem-resistant Enterobacterales; ESBL-E, extended-spectrum beta-lactamase Enterobacterales; GNR, gram-negative rods; OR, odds ratio; MDR, multidrug-resistant organism; NA, not applicable; OR, odds ratio; PsA, *Pseudomonas aeruginosa*; RR, relative risk.

### Characteristics of Systematic Reviews

Table 1 provides an overview of included studies and the main characteristics of the 17 included systematic and meta-analyses. Of these, 5 focused on ESBL-E [9, 10, 13, 16, 18], 3 only included CRE [15, 21, 23], and the remaining addressed multiple MDR-GNB [3, 11, 12, 14, 17, 19, 20, 22, 24]. All carbapenem-resistant Enterobacterales (CRE) were combined irrespective of the underlying mechanism of resistance given inconsistency in genotype reporting. Eleven studies conducted a meta-analysis [9–13, 15–17, 22–24]. All studies evaluated intestinal colonization. Six studies also evaluated respiratory colonization [11, 14, 17, 20–22], 2 urinary colonization [11, 20], and 2 skin colonization [17, 19]. Most reviews involved hospitalized adult patients including patients in the intensive care unit (ICU). Reviews and component studies varied in their surveillance strategies for microbiologic sampling, including both active surveillance and risk-based colonization assessments [9, 11, 15], and 2 studies only included general community residents [16, 18].

### Risk of Infection

The meta-analyses reported a wide range in the incidence of infection following colonization with various MDR-GNB across different populations. Four meta-analyses reported the overall incidence of infection across all included studies [9, 11, 13, 24]. Six meta-analyses included a noncolonized comparator group to evaluate the risk of infection in colonized patients [9–11, 13, 15, 17]. One study was excluded from meta-analysis due to unclear bacterial resistance profiles after attempts to clarify with study authors were unsuccessful [3].

### Risk of Infection for ESBL-E

**Figure 2.**
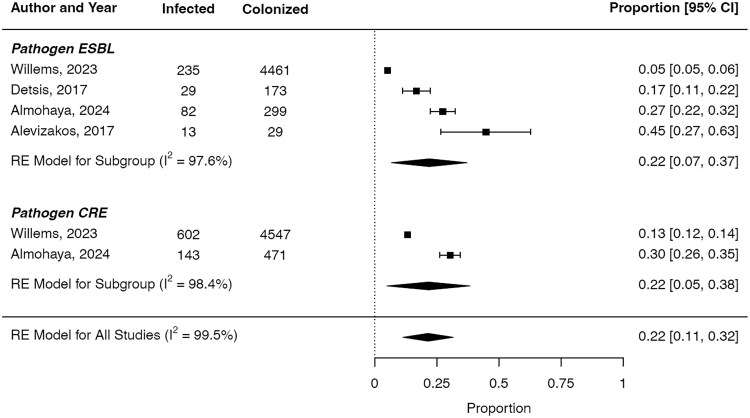
Incidence of infection after colonization with resistant Enterobacterales.

**Figure 3.**
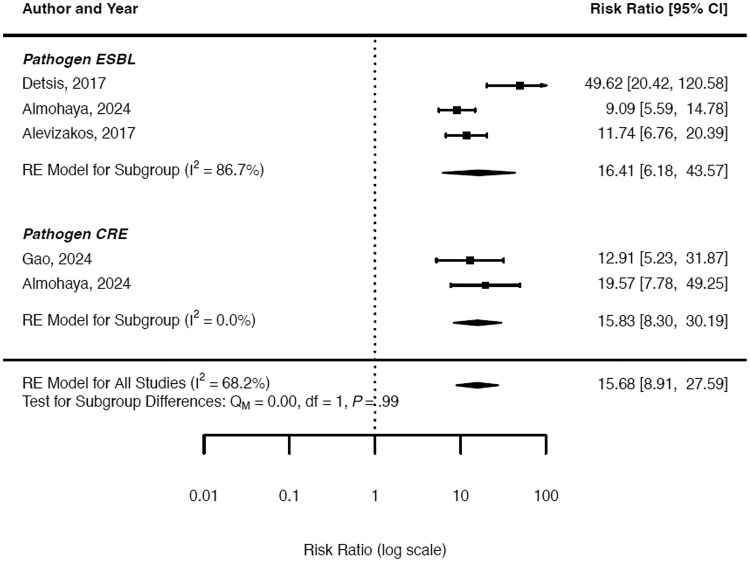
Risk of resistant Enterobacterales infection following colonization compared with noncolonization.

The incidence of infection for ESBL-E following colonization ranged from 5% in the general adult population to as high as 45% in solid organ transplant recipients [9, 24]. Across studies, the pooled incidence of infection following colonization was 22% for ESBL-E (Figure 2). The risk of infection among colonized individuals varied across studies. In solid organ transplant recipients, patients colonized with ESBL-E had a 9.09 to 11.7 times higher risk of infection compared with noncolonized individuals. In contrast, the risk of infection in adults admitted to the ICU was higher, with 1 meta-analysis reporting 50-fold increased risk compared with non-colonized patients. The overall risk ratio for infection among colonized individuals was 16.41 compared with without colonization (95% CI, 6.18–43.57) (Figure 3).

### Risk of Infection for CRE

For CRE colonization, 1 meta-analysis reported an infection incidence of 13% in adult patients, while another noted a higher incidence of 30% in solid organ transplant recipients [11, 24]. The pooled incidence of infection following colonization for CRE was 22% across the studies. (Figure 2). Only 2 studies evaluated the risk of infection associated with CRE colonization (Table 2). The risk ratio for infection following colonization with Enterobacterales was 15.83 compared with those without colonization (95% CI, 8.30–30.19) (Figure 3).

### Combined Risk of Infection for Resistant Enterobacterales

The pooled incidence of infection for both ESBL-E and CRE was 22% in colonized individuals, whereas the risk ratio for infection following colonization was 15.68 (95% CI, 8.91–27.59). In the sensitivity analysis including all studies, the corresponding pooled incidence of infection in MDR-GNB-colonized patients was 14%, with a 95% CI from 9% to 24%.

### Risk of Infection for Resistant CRAB and MDR-PsA

Only 1 review that included 1 study evaluated the risk of infection of PsA [3], and 1 review evaluated the risk association with CRAB colonization [17].

### Risk of Mortality and Transmission

The risk of mortality following infection or colonization with MDR-GNB was described in 2 reviews. Detsis et al. identified a 50% increased risk of morality associated with ESBL-E infection or colonization, while Almohaya et al. reported a >3-fold increase in the odds of mortality following CRE infection or colonization [11, 13]. Additionally, only 1 review addressed the risk of transmission and acquisition of MDR-GNB [11, 13].

### Risk Factors for Infection

Several studies highlighted risk factors associated with increased risk of infection and transmission, outlined in Table 3.

**Table 3.** Risk Factors for Infection or Transmission Following Colonization

Study	Organism	Colonization Site	Risk Factor, if Reported OR (95% CI)
Ferrer 2022 [14]	ESBL-E	Intestinal colonization	Previous penicillin use; previous cephalosporin use; previous carbapenem use; previous stay in ICU
	CR PsA	Intestinal colonization	Active exposure to antibiotic therapy; use of mechanical ventilation
		Respiratory colonization	Previous exposure to antibiotic therapy; mechanical vent at ICU admission; intake of vasopressors; presence of solid cancer
	CRAB	Intestinal colonization	Previous carbapenem use; previous cephamycin use; previous cephalosporin use; liver dysfunction
		Respiratory colonization	Previous antibiotic therapy; longer stay in ICU; use of mechanical vent; higher age; diabetes
	MDR *K. pneumoniae*	Intestinal colonization	Previous infection; previous exposure to antibiotic therapy; administration of amoxicillin-clavulanate; previous stay in ICU; longer stay in ICU; use of central venous catheter; transfer between hospital units; being in coma
		Respiratory colonization	Previous exposure to antibiotic therapy; administration of amoxicillin-clavulanate; previous infection episodes
	MDR Enterobacterales	Intestinal colonization	High-density MDR colonies; high-level resistance to carbapenems; previous stay in ICU; higher age; presence of malignancy; solid tumor; hematologic malignancy; CHF; COPD; immunologic disorder; severe liver disease; diabetes; urologic disorders; PUD; psych disorder; use of mechanical ventilation
Righi 2023 [19]	ESBL *K. pneumoniae*	…	Compared with other Enterobacterales, ESBL *K. pneumoniae* carrier status was an independent predictor of ESBL-E infection
…	CR *K. pneumoniae*	…	Carriers were more likely to develop an infection caused by CRKP compared with those who were colonized by other MDR-GNB (RR, 1.28; 95% CI, 1.04–1.58)
Wang 2024 [23]	CR *K. pneumoniae*	Intestinal colonization	ICU admission 2.59 (1.64–4.11); invasive procedure 2.53 (1.59–4.03); multisite colonization 6.24 (2.38–16.33)
Bulabula 2020 [12]	ESBL-E and MDR *Enterobacter cloacae*	…	Maternal colonization to be a risk factor for neonatal colonization in very-low-birthweight infants

Abbreviations: CHF, congestive heart failure; COPD, chronic obstructive pulmonary disease; CR, carbapenem resistant; CRAB, carbapenem resistant *Acinetobacter baumannii*; CRE, carbapenem resistant Enterobacterales; ESBL, extended spectrum beta-lactamase; ESBL-E, extended spectrum beta-lactamase Enterobacterales; GNB, Gram-negative bacteria; ICU, intensive care unit; IR, incidence ratio; KP, *Klebsiella pneumoniae*; MDR, multidrug-resistant; OR, odds ratio; PsA, *Pseudomonas aeruginosa*; PUD, peptic ulcer disease; RR, relative risk.

## DISCUSSION

This study provides a large umbrella review describing infection incidence following colonization with ESBL-E and CRE. Our work synthesizes data across multiple systematic reviews, including those limited to specific patient populations, and provides a comprehensive summary of this epidemiologically important topic. By consolidating this body of evidence, we aim to fill a critical gap in the literature, enabling a deeper understanding of the risk of infection after colonization with resistant pathogens.

The importance of this evidence is underscored by increasing attention to novel decolonization strategies for MDR-GNB [28]. The efficacy of such interventions is inherently tied to the number of infections they could potentially prevent—a calculation that requires reliable data on infection incidence following colonization. Our findings address this need by offering an evidence-based estimate that can help guide the design of interventional trials [29]. Specifically, the overall infection incidence of 22% among hospitalized patients provides a clear target for trial design, including identification of especially high-risk populations (eg, solid-organ transplant recipients) and the appropriate duration of follow-up to estimate differences in outcomes. This estimate highlights the substantial clinical impact of colonization with resistant gram-negative bacteria, emphasizing the urgency of developing and evaluating decolonization approaches, especially given the predicted increase in mortality from AMR infections in the years ahead [1].

One notable observation from this review was the consistent acknowledgment by included systematic reviews of the variable quality of the underlying primary studies. There was a general lack of standardization in definitions, methodologies, and follow-up parameters, likely contributing to heterogeneity in reported outcomes. Further, few studies provided meaningful data on the duration or burden of colonization, limiting the ability to assess its clinical significance. To strengthen the state of the literature, future cohort studies investigating infection incidence after colonization should adhere to standardized study parameters. These should include clear definitions of colonization and infection, adequate follow-up periods, and consistent microbiological and epidemiological approaches that enable standardized analytical approaches. To aid this effort, we have developed a detailed study design protocol that can facilitate high-quality and comparable research in this area, found in the Supplementary Data.

Our study has several limitations. First, there was some overlap in the primary studies included across the systematic reviews. While this may introduce some redundancy, the overlap was minimal and is unlikely to have significantly biased our estimates. Second, our results were not stratified by geography. Given that regions with a high burden of antimicrobial resistance, such as parts of Africa, Latin America, and Southeast Asia, may have different infection dynamics, additional cohort studies focusing on these underrepresented areas are essential, especially as they often have the highest burden of disease from resistant infections [30–32]. Our analysis was also limited by the relatively low number of systematic reviews on a given pathogen, especially CRE, which used only 2 available studies. The precision of the CRE estimate should be interpreted with caution. The decision to analyze studies in the aggregate may limit generalizability for specific populations at perceived higher risk of infections, such as solid-organ transplant or severe immunocompromise, and these specific population reviews can be found in Table 1. In these cases, a specific review, rather than an umbrella estimate, might provide more accurate measures of infection risk. Similarly, follow-up time was largely absent from our included studies but remains an important consideration when determining incidence rates. Third, despite specifically searching for systematic reviews addressing MDR-PsA and CRAB, we found few that met our inclusion criteria. This gap in the literature represents an area that requires urgent attention, as these pathogens contribute significantly to morbidity and mortality across health care settings and many existing decolonization strategies are of uncertain or low benefit [33]. An additional limitation is that the quality of evidence is dependent on the conduct and quality of the underlying reviews. To account for this, a sensitivity analysis was performed, including all studies regardless of quality assessment, providing an infection incidence of 14% infection after MDR-GNB colonization. Also, our reliance on the definitions of multidrug resistance provided by the included reviews potentially introduced variability, as resistance definitions were not standardized across studies. Additionally, some variability was noted in surveillance practices within reviews (including active inpatient sampling upon admission vs an individualized risk-based approach, using stool vs peri-rectal swabs, or commonly obtaining urine cultures after kidney transplantation), which may limit the generalizability depending on surveillance strategy. Finally, the decision to use an umbrella review as a literature search tool may have inadvertently excluded individual studies of importance that were more recent or not included in search terms of component reviews. To account for this, it is important to compare our point estimates with population-wide studies, which may not be feasible to conduct in most settings. We are encouraged that our estimates of infection following colonization are similar to a recently published study that took an orthogonal approach by using population-wide estimates across a health care system [34].

Importantly, the overall infection incidence of 22% among hospitalized patients colonized with ESBL-E and CRE underscores the high clinical burden associated with these pathogens. This attack rate for infections following colonization is sufficiently large to support the design of interventional studies aimed at evaluating emerging decolonization strategies. By providing an evidence-based estimate of infection incidence, our findings enable investigators to calculate effect sizes and identify optimal populations and follow-up times for these trials.

Future work should focus on employing standardized terminology and methodology in study design while addressing the geographic and pathogen-specific gaps identified in this review, which will further refine our understanding of this critical area of antimicrobial resistance. Specifically, future studies (both cohort studies and systematic reviews) should be vigilant to provide robust baseline data including matched colonized individuals with a noncolonized comparator, provide a precise description of microbiological methods used to assess resistance, quantify burden and duration of colonization when feasible, have clear follow-up intervals, and include baseline data (especially comorbidities and antibiotic exposure).

## CONCLUSIONS

To conclude, in a large systematic review and meta-analysis of systematic reviews and meta-analyses (or umbrella review), a substantial portion of those colonized with resistant gram-negative bacteria developed subsequent infection. These results can inform future development of decolonization strategies. Future observational studies should use clear terminology on type of infection, follow-up time, and type of subsequent infection to contribute to the precision of these estimates.

## Supplementary Material

ofaf365_Supplementary_Data

## Supplementary Data

Supplementary materials are available at *Open Forum Infectious Diseases* online. Consisting of data provided by the authors to benefit the reader, the posted materials are not copyedited and are the sole responsibility of the authors, so questions or comments should be addressed to the corresponding author.
