# Supplementary material for: Infection Risk Associated With Colonization by Multidrug-Resistant Gram-Negative Bacteria: An Umbrella Review and Meta-analysis
Source: Open Forum Infect Dis. 2025 Jul 2;12(7):ofaf365. doi: 10.1093/ofid/ofaf365 (PMC12273349; doi:10.1093/ofid/ofaf365)
Supplement: ofaf365_Supplementary_Data [file ofaf365_supplementary_data.docx]

**Supplemental materials**

**Search Strategies**

**Resistant Enterobacterales**

PubMed:

(("Enterobacteriaceae"[Mesh] OR Enterobacteriaceae OR Enterobacteria OR Enterobacterales OR "Escherichia coli" OR "E. coli" OR "Klebsiella pneumoniae" OR "K. pneumoniae" OR Coliform OR "Gram negative bacteria" OR "Gram-negative bacteria") AND ("Carbapenem-resistant" OR CRE OR "Carbapenemase producing" OR "Extended spectrum beta-lactamase" OR "Extended spectrum beta-lactamase producer" OR "Extended spectrum beta-lactamase producing" OR ESBL OR "ESBL producing" OR "ESBL producer")) Filters: Meta-Analysis, Systematic Review, Humans

Embase:

#1. 'enterobacteriaceae'/exp OR 'enterobacteriaceae' OR 'enteric bacteria' OR 'enterobacteria' OR 'enterobacteriacea' OR 'enterobacterium'

#2. 'carbapenem-resistant' OR cre OR 'carbapenemase producing' OR 'extended spectrum beta-lactamase' OR 'extended spectrum beta-lactamase producer' OR 'extended spectrum beta-lactamase producing' OR esbl OR 'esbl producing' OR 'esbl producer'

#3. ('enterobacteriaceae'/exp OR 'enterobacteriaceae' OR 'enteric bacteria' OR 'enterobacteria' OR 'enterobacteriacea' OR 'enterobacterium') AND ('carbapenem-resistant' OR cre OR 'carbapenemase producing' OR 'extended spectrum beta-lactamase' OR 'extended spectrum beta-lactamase producer' OR 'extended spectrum beta-lactamase producing' OR esbl OR 'esbl producing' OR 'esbl producer')

#4. ('enterobacteriaceae'/exp OR 'enterobacteriaceae' OR 'enteric bacteria' OR 'enterobacteria' OR 'enterobacteriacea' OR 'enterobacterium') AND ('carbapenem-resistant' OR cre OR 'carbapenemase producing' OR 'extended spectrum beta-lactamase' OR 'extended spectrum beta-lactamase producer' OR 'extended spectrum beta-lactamase producing' OR esbl OR 'esbl producing' OR 'esbl producer') AND ([systematic review]/lim OR [meta analysis]/lim) AND [humans]/lim

Web of Science:

1. TS=(Enterobacteriaceae OR Enterobacteriaceae OR Enterobacteria OR Enterobacterales OR "Escherichia coli" OR "E. coli" OR "Klebsiella pneumoniae" OR "K. pneumoniae" OR Coliform OR "Gram negative bacteria" OR "Gram-negative bacteria")
2. TS=("Carbapenem-resistant" OR CRE OR "Carbapenemase producing" OR "Extended spectrum beta-lactamase" OR "Extended spectrum beta-lactamase producer" OR "Extended spectrum beta-lactamase producing" OR ESBL OR "ESBL producing" OR "ESBL producer")
3. TS="Systematic Review" OR TS="Meta Analysis" OR TS="Meta-Analysis"
4. TS=human*
5. #1 AND #2
6. #5 AND #3
7. #6 AND #4

Cochrane Library:

#1 MeSH descriptor: [Enterobacteriaceae] explode all trees

#2 Enterobacteriaceae OR Enterobacteria OR Enterobacterales OR "Escherichia coli" OR "E. coli" OR "Klebsiella pneumoniae" OR "K. pneumoniae" OR Coliform OR "Gram negative bacteria" OR "Gram-negative bacteria"

#3 #1 OR #2

#4 "Carbapenem-resistant" OR CRE OR "Carbapenemase producing" OR "Extended spectrum beta-lactamase" OR "Extended spectrum beta-lactamase producer" OR "Extended spectrum beta-lactamase producing" OR ESBL OR "ESBL producing" OR "ESBL producers”

#5 #3 AND #4

Limited to Cochrane Reviews

***Pseudomonas aeruginosa***

PubMed:

(("Pseudomonas aeruginosa"[Mesh] OR "Pseudomonas aeruginosa" OR "P. aeruginosa") AND ("Drug Resistance, Multiple"[Mesh] OR "Multidrug-resistant" OR MDR OR "Extensive-drug resistant" OR "Extensively-drug resistant" OR XDR OR "Pan-drug resistant" OR PDR)) Filters: Meta-Analysis, Systematic Review, Humans

Embase:

#1. 'pseudomonas aeruginosa'/exp OR 'bacillus aeruginosus' OR 'bacillus pyocyaneus' OR 'bacterium aeruginosum' OR 'bacterium pyocyaneum' OR 'micrococcus pyocyaneus' OR 'p. aeruginosa' OR 'pseudomonas aeruginosa' OR 'pseudomonas polycolor' OR 'pseudomonas pyoceaneus' OR 'pseudomonas pyocyanea' OR 'pseudomonas pyocyaneus' OR 'blue pus organism'

#2. 'multidrug resistance'/exp OR 'mdr resistance' OR 'drug resistance, multiple' OR 'drug resistance, multiple, bacterial' OR 'drug resistance, multiple, fungal' OR 'drug resistance, multiple, viral' OR 'multi-drug resistance' OR 'multidrug resistance' OR 'multiple drug resistance'

#3. ('pseudomonas aeruginosa'/exp OR 'bacillus aeruginosus' OR 'bacillus pyocyaneus' OR

'bacterium aeruginosum' OR 'bacterium pyocyaneum' OR 'micrococcus pyocyaneus' OR 'p. aeruginosa' OR 'pseudomonas aeruginosa' OR 'pseudomonas polycolor' OR 'pseudomonas pyoceaneus' OR 'pseudomonas pyocyanea' OR 'pseudomonas pyocyaneus' OR 'blue pus organism') AND ('multidrug resistance'/exp OR 'mdr resistance' OR 'drug resistance, multiple' OR 'drug resistance, multiple, bacterial' OR 'drug resistance, multiple, fungal' OR 'drug resistance, multiple, viral' OR 'multi-drug resistance' OR 'multidrug resistance' OR 'multiple drug resistance')

#4. ('pseudomonas aeruginosa'/exp OR 'bacillus aeruginosus' OR 'bacillus pyocyaneus' OR 'bacterium aeruginosum' OR 'bacterium pyocyaneum' OR 'micrococcus pyocyaneus' OR 'p. aeruginosa' OR 'pseudomonas aeruginosa' OR 'pseudomonas polycolor' OR 'pseudomonas pyoceaneus' OR 'pseudomonas pyocyanea' OR 'pseudomonas pyocyaneus' OR 'blue pus organism') AND ('multidrug resistance'/exp OR 'mdr resistance' OR 'drug resistance, multiple' OR 'drug resistance, multiple, bacterial' OR 'drug resistance, multiple, fungal' OR 'drug resistance, multiple, viral' OR 'multi-drug resistance' OR 'multidrug resistance' OR 'multiple drug resistance') AND [humans]/lim AND ([systematic review]/lim OR [meta analysis]/lim)

Web of Science:

1.TS=("Pseudomonas aeruginosa" OR "Pseudomonas aeruginosa" OR "P. aeruginosa")

2.TS=("Drug Resistance, Multiple"[Mesh] OR "Multidrug-resistant" OR MDR OR "Extensive-drug resistant" OR "Extensively-drug resistant" OR XDR OR "Pan-drug resistant" OR PDR)

3.TS="Systematic Review" OR TS="Meta Analysis" OR TS="Meta-Analysis"

4.TS=human*

5.#1 AND #2

6.#3 AND #4

7.#5 AND #6

Cochrane Library:

#1 MeSH descriptor: [Pseudomonas aeruginosa] explode all trees

#2 "Pseudomonas aeruginosa" OR "P. aeruginosa”

#3 #1 OR #2

#4 MeSH descriptor: [Drug Resistance, Multiple] explode all trees

#5 "Multidrug-resistant" OR MDR OR "Extensive-drug resistant" OR "Extensively-drug resistant" OR XDR OR "Pan-drug resistant" OR PDR

#6 #4 OR #5

#7 #3 AND #6

Limited to Cochrane Reviews

**Carbapenem resistant *Acinetobacter baumanii***

PubMed:

(("Acinetobacter baumannii"[Mesh] OR "Acinetobacter baumannii") AND (CRAB OR "Carbapenem-resistant" OR "Carbapenemase producing")) Filters: Meta-Analysis, Systematic Review, Humans

Embase:

#1. 'acinetobacter baumannii'/exp OR 'achromobacter mucosus' OR 'acinetobacter baumanii' OR 'acinetobacter baumanni' OR 'acinetobacter baumannii' OR 'acinetobacter genomosp. 2' OR 'acinetobacter genomospecies 2' OR 'bacterium anitratum'

#2. 'crab'/exp OR crab OR 'carbapenem-resistant' OR 'carbapenemase producing'

#3. #1 AND #2

#4. #1 AND #2 AND [humans]/lim AND ([systematic review]/lim OR [meta analysis]/lim)

Web of Science:

1.TS=("Acinetobacter baumannii" OR "Acinetobacter baumannii")

2.TS=(CRAB OR "Carbapenem-resistant" OR "Carbapenemase producing")

3.TS="Systematic Review" OR TS="Meta Analysis" OR TS="Meta-Analysis"

4.TS=human*

5.#1 AND #2

6.#3 AND #4

7.#5 AND #6

Cochrane Library: #1 MeSH descriptor: [Acinetobacter baumannii] explode all trees

#2 Acinetobacter baumannii

#3 #1 OR #2

#4 CRAB OR "Carbapenem-resistant" OR "Carbapenemase producing"

#5 #3 AND #4

Limited to Cochrane Reviews

**Supplementary Table PICO Framework question**

| PICO Framework | |
| --- | --- |
| P \| Population (including setting) | Patients in all healthcare settings *without* resistant Gram-negative bacterial infection at baseline |
| I \| Intervention (Exposure) | Colonization with resistant Gram-negative bacteria (CRE, ESBL-E, MDR PsA, CRAB) |
| C \| Comparator (Non-exposure) | Absence of colonization with resistant Gram-negative bacteria (CRE, ESBL-E, MDR PsA, CRAB). |
| O \| Outcome | Risk of:   - Bloodstream infection - All infections - Mortality - Person-to-person transmission |

**Funnel plots**

Funnel Plot of Risk Ratio of Infection after colonization within included studies

**
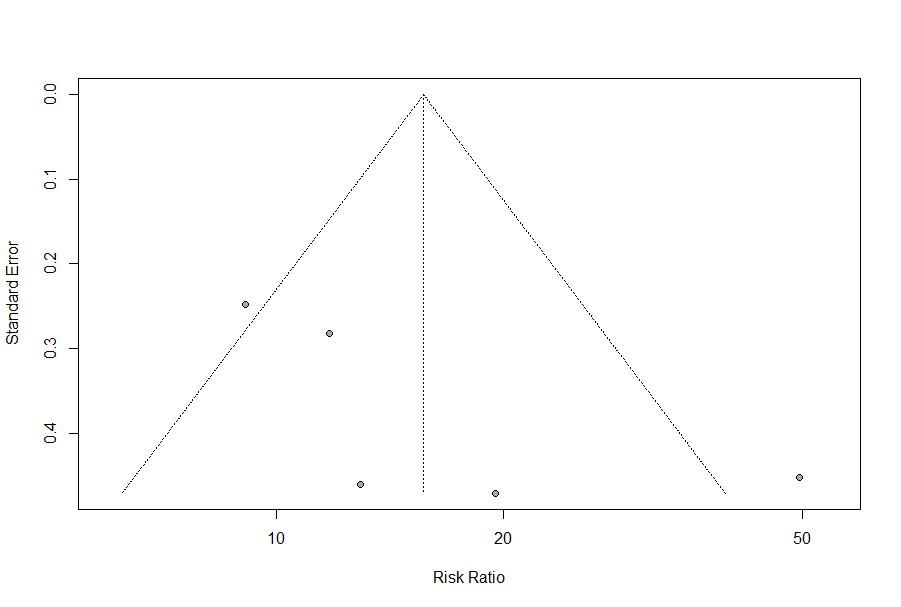
**

Funnel plot of Incidence of Infection after colonization within included studies

**
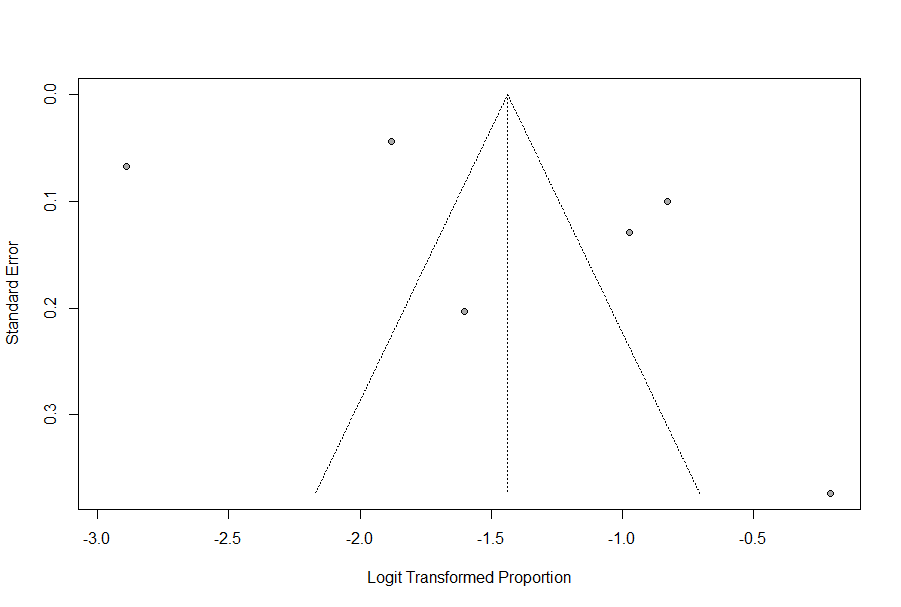
**

Funnel plot of incidence of infection after colonization for all studies (Sensitivity analysis)

**
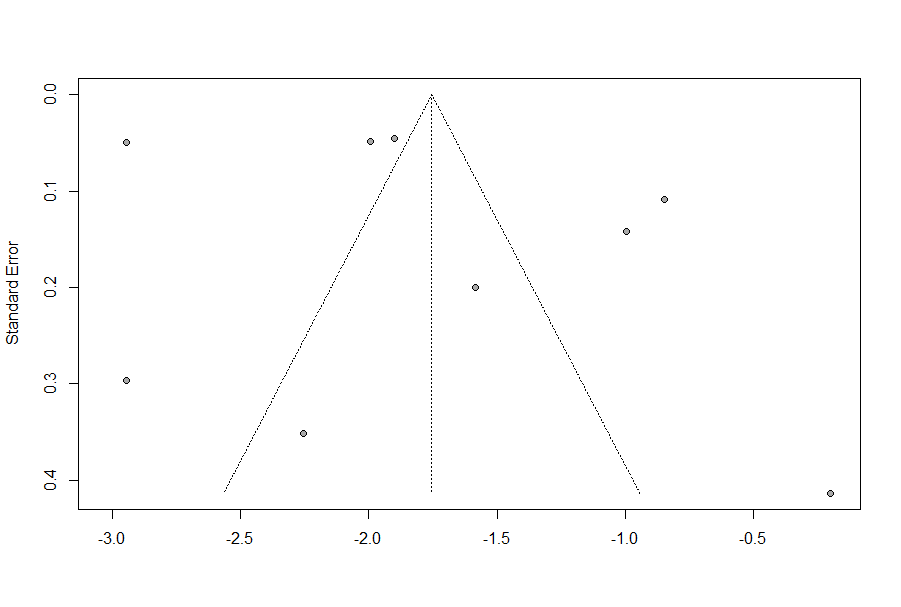
**

**Proposed Flowchart for Assessing Incidence of Infection with ESBL and Carbapenem-Resistant Gram-Negative Bacteria Following Colonization**

**1. Study Population Identification**

- **Colonized Cohort:**
  - Identify patients colonized with resistant bacteria via screening.
- **Comparator Cohort:**
  - Select non-colonized patients hospitalized in the same time frame and units.
  - When possible, match by key demographics and risk factors to control for confounding variables.

**2. Colonization Definition and Sampling Approach**

- **Microbiologic specification:** Include key data, including the precise definition of the resistance phenotype of interest, the method used for resistance testing, number of positive tests required, and the species included in the screening protocol.
- **Define Colonization by Patient Type:**
  - Non-Intubated Patients: Rectal swab.
  - Intubated Patients: Rectal + ET aspirate.
- **Colonization Definition:**
  - Colonization is defined as the presence of resistant bacteria in designated sampling sites without clinical signs of infection
  - If colonization burden is quantified, describe the method used (ex. colony forming units in urine culture; cfu/mL eSwab eluate or cfu/g of stool, normalized counts of MDRO growth on selective/differential media to total growth on non-selective media (e.g. blood agar plates))
  - **Screening Frequency:** Perform periodic colonization screenings (e.g., weekly) during the hospital stay to track changes in colonization status over time.

**3. Baseline Data Collection**

- **Data Collection for Both Cohorts:**
  - Record demographics, comorbidities, indication for hospital admission or ICU transfer, and procedural healthcare exposures.
- **Detailed Comorbidity Assessment:**
  - Include any specific comorbidity or exposure relevant to the study question
  - Use the Charlson Comorbidity Index (CCI) (or Elixhauser Comorbidity Index) to provide a standardized assessment of patient health status and adjust for comorbidities in statistical models.
- **Antibiotic Use:**
  - Record specific types and durations of antibiotic therapies received and decolonization therapies during hospitalization as potential covariates. This is especially relevant for broad-spectrum antibiotics that may influence infection risk and alter colonization status.

**4. Follow-Up and Outcome Monitoring**

- **Primary Outcome:**
  - Track the incidence of infection with the colonized resistant organism of interest.
  - Infection sources should be characterized and include, at minimum, bloodstream infections, pneumonias (especially in ventilated patients), urinary tract infections, and surgical site infections, where applicable.
  - Track time in days from exposure (confirmed colonization) to outcome (microbiologically confirmed infection).
  - Target follow-up for 90 days, with a minimum of 30 days.
- **Secondary Outcomes:**
  - Length of ICU Stay: Monitor and record for both cohorts.
  - Length of Hospital Stay: Track total duration.
  - 60-Day Mortality: Record all-cause mortality within 60 days of hospitalization.
  - In-hospital Transmission to Additional Patients: Track secondary cases to assess transmission risk.
    - When possible disclose related infection control measures used
    - Include molecular data to confirm transmission where able
  - Time to appropriate antibiotic and first appropriate antibiotic

**5. Statistical Analysis Plan**

- **Incidence Rate Calculation:**
  - Calculate infection incidence per 100 patient-days (or other metric to obtain incidence density).
- **Risk Factor Analysis:**
  - Time-to-Event Analysis: Use Cox proportional hazards modeling to assess factors influencing the time to infection post-colonization.
  - Binary Outcome Analysis: Apply logistic regression to identify risk factors associated with the likelihood of infection, including comorbidity index and antibiotic use as covariates.

**6. Ethical and Operational Considerations**

- **Informed Consent:** Obtain informed consent where necessary.
- **Site and Staff Training:**
  - Ensure personnel are trained on sample collection, handling, and data management.
- **Data Quality:**
  - Implement planned interim checks for data accuracy and completeness; communicate with clinical staff as necessary.

**7. Quality Control and Monitoring**

- **Ongoing Data Monitoring:**
  - Establish procedures for ongoing quality control of data entry and monitoring adherence to protocol.

| Sources of funding for included studies in umbrella review | |
| --- | --- |
| Study and study type | Sources of funding |
| Alevizakos 2017 | None |
| Alevizakos 2016 | None |
| Almohaya 2024 | None |
| Arzilli, 2022 | None |
| Bulabula 2020 | A.B. received doctoral stipends from Infection Control Africa Network British Society of Antimicrobial Chemotherapy (ICAN-BSAC) studentship fees, South African Medical Research Council (SAMRC), National Research Funding (NRF) |
| Detsis 2017 | NA |
| Ferrer 2022 | Shionogi S.L.U. |
| Gao 2024 | None |
| Ling 2022 | Shinogi, Merck, Pfizer funded authors outside of this work. |
| Margalit 2024 | NA |
| Martischang 2020 | R.M. was partially supported by the Swiss National Science Foundation (grant no. 407240_177454). M.E.R. was partially supported by Joint  Programming Initiative on Antimicrobial Resistance (JPIAMR) via the Swiss National Science Foundation (grant no. 40AR40-173608) |
| Righi 2023 | NA |
| Tacconelli 2019 | JTC and JRB are supported by ‘Plan Nacional de I+D+i 2013-2016’ and ‘Instituto de Salud Carlos III, Subdirección General de Redes y Centros de Investigación Cooperativa, Ministerio de Economía, Industria y Competitividad, Spanish Network for Research in Infectious Diseases (REIPI RD16/0016/0001; RD16/0016/0008)’, co-financed by the European Development Regional Fund ‘A way to achieve Europe’, Operative Programme Intelligent Growth 2014-2020. |
| Tischendorf 2016 | Supported by Agency for Healthcare Research and Quality grant No. 11670428, Department of Veterans Affairs Quality Enhancement Research Initiative 11901470, and a MERIT award from the Department of Veterans Affairs |
| Vink 2020 | None |
| Wang 2024 | None |
| Willems 2023 | The Netherlands Organization for Health Research and Development |
